# *FLAD1*‐associated multiple acyl‐CoA dehydrogenase deficiency identified by newborn screening

**DOI:** 10.1002/mgg3.915

**Published:** 2019-08-08

**Authors:** Kai Muru, Karit Reinson, Kadi Künnapas, Hardo Lilleväli, Zahra Nochi, Signe Mosegaard, Sander Pajusalu, Rikke K. J. Olsen, Katrin Õunap

**Affiliations:** ^1^ Department of Clinical Genetics, United Laboratories Tartu University Hospital Tartu Estonia; ^2^ Department of Clinical Genetics, Institute of Clinical Medicine University of Tartu Tartu Estonia; ^3^ Institute of Biomedicine and Translational Medicine University of Tartu Tartu Estonia; ^4^ Research Unit for Molecular Medicine, Department of Clinical Medicine Aarhus University Aarhus Denmark; ^5^ Department of Genetics Yale University School of Medicine New Haven CT USA

**Keywords:** *FLAD1* gene, multiple acyl‐CoA dehydrogenase deficiency, newborn screening, riboflavin

## Abstract

**Background:**

Multiple acyl‐CoA dehydrogenase deficiency (MADD), also known as glutaric aciduria type II, is a mitochondrial fatty acid oxidation disorder caused by variants in *ETFA*, *ETFB,* and *ETFDH*. Recently, riboflavin transporter genes and the mitochondrial FAD transporter gene have also been associated with MADD‐like phenotype.

**Methods:**

We present a case of MADD identified by newborn biochemical screening in a full‐term infant suggestive of both medium‐chain acyl‐CoA dehydrogenase deficiency and MADD. Urine organic acid GC/MS analysis was also concerning for both disorders. However, panel sequencing of *ETFA*, *ETFB*, *ETFDH*, and *ACADM* was unrevealing. Ultimately, a variant in the FAD synthase gene, *FLAD1* was found explaining the clinical presentation.

**Results:**

Exome sequencing identified compound heterozygous variants in *FLAD1:* NM_025207.4: c.[442C>T];[1588C>T], p.[Arg148*];[Arg530Cys]. The protein damaging effects were confirmed by Western blot. The patient remained asymptomatic and there was no clinical decompensation during the first year of life. Plasma acylcarnitine and urinary organic acid analyses normalized without any treatment. Riboflavin supplementation was started at 15 months.

**Conclusion:**

Newborn screening, designed to screen for specific treatable congenital metabolic diseases, may also lead to the diagnosis of additional, very rare metabolic disorders such as *FLAD1* deficiency. The case further illustrates that even milder forms of *FLAD1* deficiency are detectable in the asymptomatic state by newborn screening.

## 1 INTRODUCTION

Multiple acyl‐CoA dehydrogenase deficiency (MADD), also known as glutaric aciduria type II, is a mitochondrial fatty acid oxidation disorder, typically resulting from genetic defects of the electron transfer flavoprotein (ETF) or ETF ubiquinone oxidoreductase. MADD is known to be caused by variants in *ETFA*, *ETFB,* and *ETFDH*, though in recent years the riboflavin transporter genes and mitochondrial FAD transporter gene have also been associated with MADD‐like phenotype (Bosch et al., 2011; Schiff et al., 2016). In 2016, Olsen et al. described a novel form of MADD (OMIM # 255100), a potentially treatable inborn error of metabolism, caused by variations in *FLAD1*, encoding the flavin adenine dinucleotide synthase (FADS) (Olsen et al., 2016). Presently at least 13 patients have been described with variable clinical expression (Auranen et al., 2017; Garcia‐Villoria et al., 2018; Olsen et al., 2016; Ryder et al., 2019; Yildiz et al., 2018). These patients may benefit from treatment with high doses of riboflavin, and early detection is therefore important. However, there is very limited knowledge on how well patients with *FLAD1* variants are detected by newborn screening for acylcarnitines in dried blood spots. We therefore describe a patient detected via our newborn screening program with suspicion of both medium‐chain acyl‐CoA dehydrogenase deficiency (MCADD) and MADD who was ultimately found to have *FLAD1*‐associated MADD.

## 2 CASE REPORT

The patient was born following an uneventful pregnancy to a G1P0 to one mother. She was born at term with birth weight 3,134 g, length 48 cm and Apgar scores of 9 and 9 at 1 and 5 min, respectively. She had a normal postnatal course and was discharged from the birth hospital at 2 days of age. Due to positive newborn screening suspicious for MCADD or MADD, she was brought to clinic for additional investigations. At the first visit on day of life 15, her weight was 3,090 g and she had mild feeding problems; lactation consultation was provided. On day of life 20, an echocardiogram was normal. Blood acylcarnitine profile and urinary organic acid analysis revealed concerning for possible MCADD and MADD (Table 1). As the infant remained asymptomatic, the decision was made not to add any specific medication and to shorten the breastfeeding intervals. The family was advised to seek immediate medical attention if any complaints or symptoms like lethargy, vomiting, and/or hypotonia arose.

**Table 1:** Newborn screening and serum acylcarnitine analyses results

	Newborn screening (reference range) µmol/L	Serum acylcarnitine analysis (reference range) µmol/L	Serum acylcarnitine analysis (reference range) µmol/L
age	72 hr	15 days	3 months
C0	23.26 (7.97–55.6)	28.75 (10–60)	33.0 (10–60)
C4	**1.33 (<0.87)**	**1.15 (0.03–0.79)**	0.4 (0.03–0.79)
C5	**0.6 (<0.5)**	0.36 (<0.44)	0.3 (<0.44)
C5DC	0.29 (<0.51)	**0.43 (0.03–0.29)**	0.15 (0.03–0.29)
C6	**0.39 (<0.36)**	**0.52 (<0.18)**	0.14 (<0.18)
C8	**0.43 (<0.33)**	**1.07 (<0.31)**	0.27 (<0.31)
C10	**0.43 (<0.32)**	**1.0 (0.01–0.51)**	0.24 (0.01–0.51)
C10:1	**0.11 (<0.05)**	0.18 (0.01–0.21)	0.07 (0.01–0.21)
C12	0.31 (<0.57)	**0.34 (0.01–0.19)**	0.11 (0.01–0.19)

Note

Out‐of‐range results are indicated by bold text.

In addition, acute illness protocols typically used for MCADD were provided to the family. During the next visits at the age of 3 and 7 months, parents did not have any complaints about the infants feeding, health, or development. During the first year of life, she had no viral or bacterial infections and no decompensations. Her development has been age appropriate. Exome sequencing (ES) was performed to clarify the aetiology of metabolic screening alterations. At the age of 15 months, after ES and consulting the parents, treatment with vitamin B2 (100 mg/day) was initiated.

## 3 MATERIALS AND METHODS

Written informed consent was obtained from the family for the routine clinical study.

### 3.1 Biochemical analysis

Acylcarnitine analyses from dried blood spot and serum were performed by tandem mass spectrometry (Xevo TQD Triple Quadrupole Mass Spectrometer [Waters]). Organic acids from urine were measured by gas chromatography mass spectrometry (GC/MS) (Agilent 7890B GC with 977A MSD running on MassHunter software [Agilent Technologies]). All used reference intervals were age‐specific and based on the previous experience of the laboratory (Reinson et al., 2018).

### 3.2 Molecular genetic analysis

As an initial genetic test, the TruSight One panel (Illumina, 4,813 genes) was sequenced to screen for disease causing variants *ETFA*, *ETFB*, *ETFDH,* or *ACADM* in a metabolic disease panel (670 genes).

Proband‐only ES was performed in clinical diagnostic settings at Tartu University Hospital to search for genetic variants in other disease‐associated genes. The exome was enriched using SureSelect Human All Exon V5 kit (Agilent), and sequenced on a HiSeq 4000 (Illumina) platform. The data processing and variant calling pipeline followed Genome Analysis Toolkit's best practice guidelines (Van der Auwera et al., 2013) and the specifics of our in‐house pipeline have been previously described (Pajusalu, Reimand, & Ounap, 2015). Sanger sequencing was used for the confirmation of detected variants.

### 3.3 Western blotting

Dermal fibroblast culturing and protein extraction methods have been previously described (Ryder et al., 2019). Twenty and 40 µg of the total cell protein extract, for the flavoproteins and FADS, respectively, (determined by the Bradford Protein assay [Bio‐Rad]) were analyzed by SDS–PAGE on Criterion™ TGX Stain‐free™ Precast Gels (any kD) (Bio‐Rad) in Tris‐Glycine 0.1% SDS buffer. All Blue Standards (Bio‐Rad) were used as molecular weight (MW) marker. Proteins were blotted onto PVDF membranes (midi format, 0.2 µm [Bio‐Rad]) by semidry electroblotting (Trans‐Blot^®^ Turbo^™^ Transfer System [Bio‐Rad]) for 30 min. The PVDF membranes were incubated 1 hr in 5% nonfat skim milk (VWR). Transferred proteins were incubated overnight with primary polyclonal rabbit antibodies: anti‐very long‐chain acyl‐CoA dehydrogenase (VLCAD) antibody (kindly provided by Dr. Arnie Strauss), diluted 1:10,000 (detected at MW 68 kDa), anti‐short‐chain acyl‐CoA dehydrogenase (SCAD) antibody (kindly provided by Dr. Arnie Strauss), diluted 1:15,000 (detected at MW 40 kDa), anti‐ETF A & B (ETF α & β) antibody (kindly provided by Dr. Kay Tanaka) diluted 1:20,000 (detected at MW 32 & 27 kDa), and anti‐FLAD1 antibody (HPA028563) (Sigma Aldrich), diluted 1:250 (detected at MW 50 and 26 kDa). Polyclonal goat anti‐rabbit HRP antibody (DAKO) at dilution 1:20,000, for the FADS and 1:25,000 for the flavoproteins blotting, were used as secondary antibody. ECL plus Western Blotting Detection System (Amersham Biosciences) was used for protein detection, according to manufacturer's recommendations. Detection of proteins was performed using the ImageQuant LAS 4000 (GE Healthcare). The intensities of bands were quantified using ImageQuant TL (GE Healthcare) and normalized to the total protein content.

## 4 RESULTS

### 4.1 Biochemical results

The newborn screening sample was obtained at 73 hr of life and revealed slightly elevated levels of butyryl‐(C4), isovaleryl‐(C5), hexanoyl‐(C6) octanoyl‐(C8), decanoyl‐(C10), and decenoylcarnitine (C10:1) (Table 1). Results obtained from newborn screening in Estonia are reported with significant deviations from reference values from the Collaborative Laboratory Integrated Reports database, a tool created by the Mayo Clinic's Biochemical Genetics Laboratory. This data analysis was consistent with a possible diagnosis of MCADD and/or MADD. The first blood acylcarnitine analysis performed at the age of 15 days revealed an increased amount of C4, glutaryl‐(C5DC), C6, C8, C10, and dodecanoylcarnitine (C12) (Table 1). The two highest peaks in the acylcarnitine profile were C8 (344 m/z) and C10 (372 m/z), which were most suggestive of MCADD, but MADD could not be excluded. The urine organic acid GC/MS analysis revealed elevated excretion of adipic, suberic, ethylmalonic, glutaric, 2‐OH‐glutaric and sebacic acid; small amounts of 5‐hydroxyhexonic, 3‐OH‐adipic, and 3‐OH‐sebacic acids were also seen, which could also be consistent with MCADD versus MADD. At the age of 3 months, blood acylcarnitine analysis revealed no abnormalities and the free carnitine level was also normal, excluding carnitine deficiency as an explanation for the normal blood acylcarnitine profile (Table 1). Additionally, the urine organic acid GC/MS analysis profile showed persistent excretion of ethylmalonic, sebacic and 2‐OH glutaric acid, consistent with MADD. After the age of 12 months, repeated acylcarnitine and urinary organic acid analyses revealed no abnormalities. This normalization of the biochemical phenotype was achieved without riboflavin treatment. Only the creatine kinase remained slightly elevated (330 U/L; ref.range <228 U/L) in serum.

### 4.2 Molecular genetic results

Panel sequencing revealed no pathogenic variants in the classical MADD genes (*ETFA*, *ETFB,* and *ETFDH),* and in the MCADD gene (*ACADM*). ES revealed a compound heterozygous variant in *FLAD1*: NM_025207.4: c.[442C>T];[1588C>T], p.[Arg148*];[Arg530Cys]. The latter variant has been described previously (Auranen et al., 2017; Olsen et al., 2016). Sanger sequencing in the index patient confirmed the molecular diagnosis, showing that the first variant was inherited from the mother and the latter from the father. Reported variants have been submitted to the Global Variome shared Leiden Open Variation Database (patient ID: 00230572).

### 4.3 FADS and flavoproteins levels in fibroblasts

**Figure 1:**
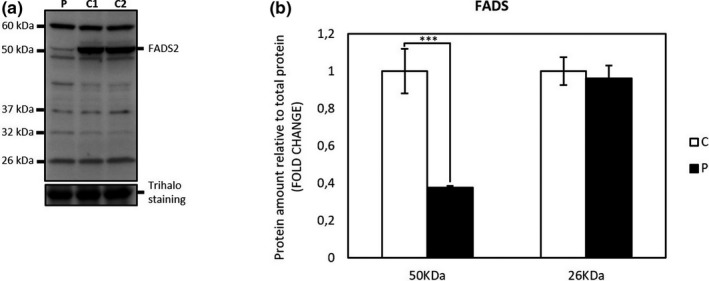
(a) Representative immunoblot analysis of FADS Proteins. Protein extracts from cultured human dermal fibroblasts were separated by SDS‐PAGE and immunoblotted with a polyclonal antibody, raised against the C‐terminal part of human cytosolic FAD synthase protein (FADS2). The patient cells were cultured in three separate cultures together with two healthy control individuals (C1 and C2), protein was extracted and 40 µg loaded on Criterion™ TGX Stain‐free™ Precast Gels (any KD) (Bio‐Rad). (b) Protein intensities were quantified relative to total protein content (Trihalo staining). Quantification of patient FADS relative to combined two control individuals as shown. The error bars represent standard error of mean (SEM) of three independent experiments, student's *t* test: ***p < .001. FADS, flavin adenine dinucleotide synthase

**Figure 2:**
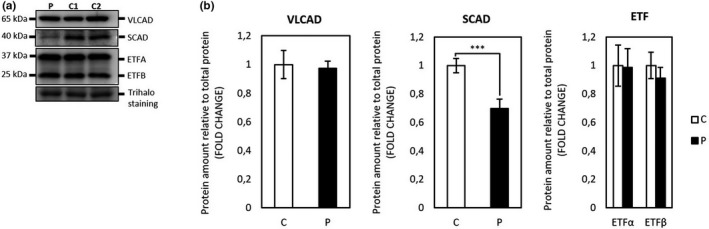
(a) Representative immunoblot analysis of selected mitochondrial flavoproteins. Protein extracts from cultured human dermal fibroblasts, were loaded in 20 μg. Samples were separated by SDS‐PAGE and immunoblotted with polyclonal antibodies raised against each of the four mitochondrial flavoproteins VLCAD, SCAD or the two ETF subunit proteins. The patient cells were cultured in three separate cultures together with two healthy control individuals (C1 and C2), protein was extracted and loaded on Criterion^™^ TGX Stain‐free^™^ Precast Gels (any kD) (Bio‐Rad). (b) Protein intensities were quantified relative to total protein content (Trihalo staining). Quantification of patient proteins relative to combined two control individuals as shown. The error bars represent standard error of mean (SEM) of three independent experiments, student's *t* test: ***p < .001. SCAD, short‐chain acyl‐CoA dehydrogenase; VLCAD, very long‐chain acyl‐CoA dehydrogenase

Flavin adenine dinucleotide synthase and flavoproteins levels in patient and control fibroblasts were determined by Western blot analysis. As expected from the *FLAD1* genotype, the patient fibroblasts displayed significantly decreased amount of the cytosolic full‐length 50 kDa FADS protein compared to control fibroblasts (student's *t* test *p* < .001). However, the 26 kDa FADS band, containing an intact and functional FADS domain (Leone et al., 2018; Olsen et al., 2016), seems equally expressed in both patient and control fibroblasts (Figure 1a,b). Mitochondrial flavoproteins comprising VLCAD, and the two ETF subunit proteins showed no difference in the patient as compared to controls. However, SCAD was significantly decreased in the patient fibroblasts as compared to the control fibroblasts (student's *t* test *p* < .001) (Figure 2a,b).

## 5 DISCUSSION

We here describe a case of *FLAD1*‐associated MADD diagnosed after a positive newborn screening result. In Estonia, newborn screening for phenylketonuria was introduced in 1993 (Ounap, Lillevali, Metspalu, & Lipping‐Sitska, 1998), and in 1996 screening for congenital hypothyroidism was added. In 2014, expanded neonatal screening was initiated and presently includes 19 treatable congenital metabolic diseases (Reinson et al., 2018). In this particular case, the abnormal newborn screening result was concerning for MCADD and/or MADD given the modest elevation of C4‐C10. These findings were similar to the case described by Ryder et al., who reported elevation of C6‐C12 in a newborn, who 8 years later was confirmed to have a homozygous *FLAD1* variation c.[745C>T], p.[Arg249*] (Ryder et al., 2019). Confirmatory urine organic acid GC/MS analysis of our patient revealed findings for both MCADD and MADD, however, gene panel sequencing did not confirm either MCADD or classical MADD. Short feeding intervals and contingency planning for management in the setting of acute illness as would be done for MCADD and MADD was recommended, but no carnitine or riboflavin trial treatment were initiated. The patient was closely monitored through first year of life and did not have any episodes of illnesses or documented hypoglycemia. Her neurologic exam also remained normal. Unlike the biochemical aberrances found in the patient described by Ryder et al., the biochemical markers of our patient normalized without specific treatment (Ryder et al., 2019).

ES revealed compound heterozygosity in *FLAD1* encoding the FADS (EC 2.7.7.2). Pathogenic *FLAD1* variants have been shown to cause of a novel form of MADD (Olsen et al., 2016). The missense NM_025207.4: c.[1588C>T], p.[Arg530Cys] variant in the FADS domain, as found in our patient, has been previously reported to give rise to an unstable protein with reduced FADS activity in a patient with a mild and late‐onset phenotype (Auranen et al., 2017; Olsen et al., 2016). The nonsense NM_025207.4: c.[442C>T], p.[Arg148*] variant, located in exon 2 is novel. It has been shown that nonsense variations in exon 2 may result in some residual FADS activity because of the existence of a FADS isoform that lacks exon 2, but has an intact and functional FADS domain (Olsen et al., 2016). The protein‐damaging effect of the *FLAD1* genotype in our patient was confirmed by Western blot of the FADS protein, which showed a significantly decreased amount of the cytosolic full‐length 50 kDa FADS protein compared to control fibroblasts. However, the 26 kDa FADS band, containing the truncated FADS isoform with an intact and functional FADS domain (Leone et al., 2018; Olsen et al., 2016), seems equally expressed in both patient and control fibroblasts (Figure 1a,b). Additionally, among the mitochondrial flavoproteins only SCAD was significantly decreased in the patient fibroblasts as compared to the control fibroblasts (Figure 2a,b). The clear decrease in the amount of SCAD could be explained by the fact that SCAD protein turnover in particular as compared to the other acyl‐CoA dehydrogenases is highly dependent on FAD as a cofactor (Lucas et al., 2011).

Based on the *FLAD1* genotype, we assume that our patient might develop a milder phenotype (lipid storage myopathy), similar to previously described patients, who are compound heterozygous with the NM_025207.4: [1588C>T], p.[Arg530Cys] variant in one allele and an exon 2 nonsense variant in the other allele (Auranen et al., 2017; Olsen et al., 2016). One of those patients first presented clinically at the age of 20 years, although since childhood she had become symptomatic after prolonged exertion (Auranen et al., 2017). A second patient presented with the first symptoms at the age of 44 years with presumably no obvious clinical symptoms during childhood and adolescence (Olsen et al., 2016). This milder phenotype in our patient is supported by the absence of clinical symptoms at the age of 18 months and only mildly increased CK 330 U/L (ref. range <228 U/L) at the age of 14 months. Even though she remained asymptomatic at the age of 15 months, we suggested to start riboflavin treatment (100 mg/day), due to the possible benefits and low risk for adverse effects.

## 6 CONCLUSION

Newborn screening is designed to screen for specific treatable congenital metabolic diseases, though due to the use of tandem MS technology has the potential to also detect very rare metabolic disorders that are not the intended targets of the newborn screening assay. One example of such a disorder is MADD, which may be caused by biallelic *FLAD1* variants. This is an important diagnosis to make in the newborn period, as it might be riboflavin responsive and treatable. The present case therefore illustrates that *FLAD1* genotypes associated with a mild and late‐onset disease can be detected and potentially prevented by early newborn screening.

## CONFLICT OF INTEREST

The authors declare no conflict of interest.
